# Fluoride and Calcium Release from Alkasite and Glass Ionomer Restorative Dental Materials: In Vitro Study

**DOI:** 10.3390/jfb14020109

**Published:** 2023-02-15

**Authors:** Alessandro Di Lauro, Fabiana Di Duca, Paolo Montuori, Amanda Maria de Oliveira Dal Piva, João Paulo Mendes Tribst, Alexandre Luiz Souto Borges, Pietro Ausiello

**Affiliations:** 1 Department of Neurosciences, Reproductive and Odontostomatological Sciences, University of Naples Federico II, Via S. Pansini 5, 80131 Naples, Italy; 2 Department of Public Health, University of Naples Federico II, Via Sergio Pansini 5, 80131 Naples, Italy; 3 Department of Dental Materials, Academic Centre for Dentistry Amsterdam (ACTA), University of Amsterdam and Vrije Universiteit Amsterdam, 1081 LA Amsterdam, The Netherlands; 4 Department of Oral Regenerative Medicine, Academic Centre for Dentistry Amsterdam (ACTA), Universiteit van Amsterdam and Vrije Universiteit Amsterdam, 1081 LA Amsterdam, The Netherlands; 5 Department of Dental Materials and Prosthodontics, Institute of Science and Technology, São Paulo State University (Unesp), São José dos Campos 12245-000, Brazil

**Keywords:** fluorides, calcium, ions, glass ionomer cement, alkasite, dental materials

## Abstract

This study evaluated the effect of pH and temperature on the ion (F^−^ and Ca^2+^) release of a resin-based material containing alkaline fillers and a self-setting high-viscous glass ionomer cement. Disks were prepared according to manufacturers’ instructions for both materials: the EF group (Equia Forte HT filling, GC) and the CN group (*Cention N*, Ivoclar). Specimens were immersed in 50 mL buffer solution with three different pHs (4.8, 6.8, and 8.8), and stored at 0°, 18°, 37°, and 44 °C. After 24 h, 7 d, and 28 d, cumulative F^−^ and Ca^2+^ releases were analyzed by chromatography and mass spectrometry, and pH was measured. Both materials showed minimal changes in pH with final values after 28 d of 5.17 ± 0.56 for CN and 5.12 ± 0.24 for EF. In all experimental conditions, the percentages of ion release were higher for EF than for CF. In particular, both materials showed a significant difference in temperature in F^−^ release. Regardless of the pH values, the highest Ca^2+^ ion release was after 28 days, with a significant difference in temperature for CN and EF. Within the limit of this study, the temperature storage influenced ion release and the high-viscous glass ionomer showed the maximum values.

## 1. Introduction

Restorative dentistry therapies are essentially based on the use of indirect or direct techniques and materials. The clinical aim is to partially or fully restore the dental masticatory function due to decayed or missing teeth [1,2,3]. Resin-based filling dental composite materials are polymer compounds. They represent the first choice among filling materials for the direct restoration of decayed teeth by dentists [4]. They need a bulk or multilayer direct adhesive application technique on dental tissues and are part of the larger class of shrinking polymer compounds. The restorative dental filling alternatives are represented by glass ionomer cements (GICs), resin-modified glass ionomer cements, or compomers [5]. GICs are derived by an acid-base reaction and their wear resistance ability is reduced over time. Therefore, they have indications only in limited clinical cases, when the incidence of masticatory fatiguing loading on teeth is limited in time, such as in primary dentition therapy [6,7]. Notwithstanding the selected restorative material, the risk of recurrent caries in teeth that received a resin polymeric composite-based restoration is a well-documented condition. Secondary caries is widely associated with multifactor conditions [8]. One of them is the polymerization shrinkage stress of these dental materials used to fill decayed teeth and the debonding of the materials from the dental walls, facilitating gap formation. This physical phenomenon still represents the most difficult clinical step to control in the management of polymeric filling materials by the dentist [9].

The interfacial gap means micro-leakage at the margins of the restored tooth, leading to the progression of bacteria between the restoration and the dental substrates, enamel, and dentin. In some cases, the biofilm formation promotes a local pH drop as a consequence of acid production, reducing the saliva buffer effect and leading to enamel demineralization. In fact, in a highly acidic environment, the natural remineralization process operated by saliva neutralization is insufficient for achieving enamel remineralization. This chronic process induces caries over time [10]. However, the acid pH can be prevented through ions released, commonly derived from enamel hydroxyapatite [11], in the microenviroment. Al-Qarni et al. advised that a pH of 6.0 is a safe zone with a low risk of dental caries; however, a pH of 5.5 to 6.0 is a potentially cariogenic zone while a pH of 4.0 to 5.5 is a high-risk zone for incidence of dental caries [12]. Simeonov et al. showed that remineralization through mineral redeposition in surface defects is possible through calcium and phosphate release [13].

Therefore, a new dental filling material alternative to polymeric curing filling should be able, on the one hand, to resist the local pH variation and, on the other hand, to neutralize acid and bacteria biofilm around dental restorations by ion release such as F^−^ and Ca^2+^ [14].

During the past years, bioactive restorative materials [13,14] such as glass ionomer cements (GICs) and later resin-modified glass ionomers were offered on the market. These materials provided an enamel demineralization resistance effect based on fluoride release [15]. Nevertheless, their mechanical strength was relatively lower than that of composite resin-based materials [16]. So, they were not indicated as definitive dental restorations. To overcome the mechanical drawbacks of GICs and the polymer shrinkage of the resin-based materials [17,18], new dental materials were recently developed to be used as long-term restorative materials: a dual-cured, low resin-based material containing alkaline ion (CaF_2_, SiO_2_, CaO, and Na_2_O, forming OH^−^ groups upon reaction with water and releasing Ca^2+^) named Cention N, and a self-curing resin-free highly viscous glass ionomer cement (F^−^ and Ca^2+^ releasing) named Equia Forte HT were presented. These restorative dental filling materials are intended to restore teeth, allowing the remineralization of enamel and dentin in the initial caries process by pH modification [17]. The objective of this investigation was to assess the F^−^ and Ca^2+^ releasing at different pH and temperatures (T, °C). The null hypothesis is that there are no differences in the percentage of ions released by both restorative filling materials and that the pH and T values of the environment do not influence it.

## 2. Materials and Methods

### 2.1. pH Measurements, Fluoride Ion Release, and Calcium Concentration

In this study, two types of commercially available restorative materials were investigated. The selected types and compositions of material are summarized in Table 1. Dental materials specimens were prepared according to the manufacturers’ instructions: by light curing for CN (Cention N, Ivoclar, Linchstein, lot n. Z037KP) or self-reaction modalities for EF (*Equia Forte HT filling*, GC, Japan, lot n. 211122A). Briefly, for CN the specimens were prepared using a stainless-steel cylinder with a 10 mm diameter and 2 mm thickness. For EF samples were prepared using a Teflon cylinder with a 10 mm diameter and 2 mm thickness. Each specimen was mixed by a 3M^TM^ ESPE^TM^ CapMix^TM^ mixer (3M ESPE, Seefeld, Germany) for 10 s and immediately applied to the cylinders. The samples were gently compressed with a celluloid strip and a smooth condenser to prevent the formation of air bubbles and to obtain a smooth and flat surface. No coating varnish was applied at the top. After 5 min, they were removed and the surface was polished with 800 grit abrasive paper using a water-cooled rotating polishing machine (Ecomet 30, Buehler Ltd., Lake Bluff, IL, USA). Therefore, samples of both materials (n = 5 for each material), were immersed in 50 mL of buffer solution in three different pH environments (4.8, 6.8, and 8.8), stored in a refrigerator at 0 °C, and in thermostatically controlled laboratory ovens (Precision Thelco, Thermo Fisher Scientific, Waltham, MA, USA) at 18 °C, 37 °C, and 44 °C. For the acid environment, a 1M acetic acid/sodium acetate buffer solution (CH_3_COOH/CH_3_COONa_3_H_2_O) was prepared at pH = 4.8; for the neutral environment, a Phosphate-Citrate buffer solution was used at pH = 6.8. This buffer solution was prepared using sodium hydrogen phosphate (Na_2_HPO_4_) and citric acid (C_6_H_8_O_7_·H_2_O). Moreover, for the basic environment at pH = 8.8, a 1M Tris(hydroxymethyl)amino methane hydrochloride (Tris-HCl) buffer solution was prepared using Tris (C_4_H_11_NO_3_) and hydrochloric acid (HCl). After 24 h, 7 days, and 28 days of soaking in water, in which the materials were immersed, they were transferred to a 50 mL falcon and analyzed. Cumulative fluoride and calcium ion releases and change in pH were assessed at the end of 7 days, 14 days, and 28 days by ion chromatography, mass spectrometry with inductively coupled plasma, and pH measurements using a digital pH meter.

For pH measurements, 5 mL of soaking water was separated from the sample and transferred to a 15 mL falcon. A digital pH meter (Mettler Toledo-SevenExcellence pH/Cond meter S470-Std-K), previously calibrated with standard solutions, was used for pH measurement. For fluoride (F^−^) release, each sample in 1 mL of soaking water was transferred to a 1.5 mL vial, and the F^−^ amount was measured using a DIONEX Integrion HPIC^TM^ ICS1100 ion chromatography system (Thermofisher). The Dionex 1100 was equipped with a Dionex EGC 500 KOH RFIC^TM^, potassium hydroxide (KOH) eluent generator cartridge, and an IonPac AS27 RFIC^TM^ (4 × 250 mm) (Thermofisher) analytical column. Deionized water (>18 MΩ) was used to generate the eluent [19,20]. A total of 25 µL of each sample was injected into the injection loop of the instrument and a flow rate of 1.0 mL/min was used. The fluoride concentration was determined on the basis of the retention time and the area of the corresponding chromatographic peak, by interpolation with the calibration line, prepared by standard solutions of the analysis. For the evaluation of calcium (Ca^2+^), samples (soaked in 10 mL of water) were acidified with 1% HNO_3_/HCl (3:1% *v*/*v*) and analyzed using a trace elemental analyzer (Thermo Scientific TM ICAP TM RQ) and an inductively coupled plasma mass spectrometer (Q-ICP-MS), operated by software (Qtegra^TM^). The operating conditions of the equipment (Q-ICP-MS) were optimized using a tuning solution (Ba, Bi, Ce, Co, In, Li, U 1.00 µg/L, Thermo Scientific). The analyses were performed in KED (Kinetic Energy Discrimination) mode using Helium as collision gas. The concentrations of the analyses were estimated by calibration line (CertiPUR^®^, Merck, Darmstadt, Germany) (r^2^ > 0.98) [21,22,23,24].

### 2.2. Statistical Analysis

Descriptive statistics including the mean, standard error, minimum, and maximum values were evaluated for all specimens. Statistical analyses of the results were performed using the Stata 14.0 program (College Station, TX, USA). Shapiro–Francia W’ tests were used to assess the normality of the data distributions followed by the Doornik–Hansen test for multivariate normality. The level of significance was set to 0.05.

## 3. Results

### 3.1. pH Measurements

Table 2 and Figure S1 illustrate the pH variation following the immersion of the materials in buffer solutions at acid (4.8), neutral (6.8), and basic pH (8.8), respectively. The curve of both materials showed minimal changes in pH with final values after 28 days of 5.17 ± 0.56 for CN and 5.12 ± 0.24 for EF. Moreover, CN and EF were not statistically significant in terms of pH variation (*p*-value > 0.05). In an acidic environment, pH values were observed to increase after 7 days and then decrease until 28 days. In a neutral environment, the pH decreased by one unit in the first 24 h of immersion (pH = 5.67 and 5.77, for CN and EF, respectively), and then returned to a situation like the initial after 7 and 28 days. In an alkaline medium, the pH decreased by one unit and remained constant throughout the evaluated periods. The slightly higher pH variation at high temperatures could be considered minimal since, even if the pH increases, its increase is at most one unit; therefore, maintaining the conditions of acidity, neutrality, and basicity in the scenarios considered. Thus, the minimal pH variations still provided evidence of the buffer effect of the material.

### 3.2. Fluoride Ion Release

Table 3 and Figure S2 show the results obtained for the release of fluoride ions for both evaluated materials. Although differences in the amounts of fluoride released from the materials were found, the pattern was similar in various media. The results of linear multiple regression indicated that CN and EF showed a significant difference by temperature in fluoride ion release (*p*-value < 0.05) for both materials, but there was a significant dependence on pH value only for CN (*p*-value = 0.007), not for EF (*p*-value = 0.508). In fact, in both materials, for all three pH values, the highest concentrations were recorded after 28 days. For CN, fluoride ion concentrations were in the range of 0.15–10.08 mg/L. The maximum amounts detected were 5.27 ± 0.37 mg/L, 7.95 ± 0.69 mg/L, and 10.08 ± 0.61 mg/L at pHs of 4.8, 6.8, and 8.8, respectively, and 44 °C. For EF, fluoride ion concentrations were in the range of 0.11–32.56 mg/L. The highest fluoride ion release was observed after 28 days at 37 °C in all three pH conditions of the study. Specifically, at pH 4.8, the release was 28.71 ± 1.12 mg/L and 29.62 ± 0.66 mg/L after 7 and 28 days, respectively. In a neutral environment (pH 6.8), the highest fluoride concentrations were 16.16 ± 0.63 mg/L (37 °C) and 15.94 ± 0.91 mg/L (44 °C). In the basic environment (pH = 8.8), the fluoride concentration found at 44 °C had doubled (28.62 ± 1.18 mg/L) after 21 days compared to the value recorded after 7 days (14.86 ± 0.26 mg/L). However, in the basic environment, the maximum fluoride ion release was detected at 37 °C (32.57 ± 1.11 mg/L).

### 3.3. Calcium Concentration

The results of the calcium ion concentrations for both materials are shown in Table 4 and Figure S3. Regardless of the group, for all three pH values, the highest amount of calcium ions was detected after 28 days. The results of multiple linear regression showed that CN and EF had a significant difference in temperature in calcium ion release (*p* < 0.05), but there was no significant difference in pH variation (*p*-value = 0.688 for CN and *p*-value = 0.144 for EF). For CF, calcium ion concentrations were in the range of 0.13–14.31 mg/L. In an acid environment, the highest concentrations were found at 37 °C after 7 (12.58 ± 0.43 mg/L) and 28 days (14.31 ± 0.74 mg/L), respectively. The amounts found in a neutral and basic environment, on the contrary, were lower than those mentioned above. In particular, the minimum concentrations were found at 0 °C for all three study times. However, the highest amount detected at pH = 6.8 was 6.22 ± 0.17 mg/L at 44 °C and 6.30 ± 0.47 mg/L at 37 °C after 7 and 28 days, respectively. For pH = 8.8, high concentration values were found at 44 °C after 7 (12.94 ± 0.59 mg/L) and 28 days (13.35 ± 0.76 mg/L). For EF, calcium ion concentrations were in the range of 0.27–29.57 mg/L. The highest amount of calcium ions was observed after 28 days in the three pH conditions of the study. In detail, at pH 4.8, the maximum value was recorded at 44 °C (22.99 ± 0.66 mg/L), but the value was comparable with that found at the same temperature after 7 days (21.64 ± 0.67 mg/L). In a neutral environment (pH 6.8), the amount of calcium ions increased from 25.94 ± 0.84 mg/L to 29.57 ± 0.60 mg/L at 37 °C after 7 and 28 days, respectively. Lastly, in the basic environment (pH 8.8), the largest increases were observed after 7 days from the beginning of the experiment at 37 °C, from 2.33 ± 0.26 mg/L (after 1 day) to 11.36 ± 0.81 mg/L, remaining almost constant after 28 days (11.88 ± 0.91 mg/L).

## 4. Discussion

The objective to remineralize initial enamel lesions in proximal adjacent surfaces, especially in high-risk caries patients, is part of contemporary dentistry [25]. Therefore, the biofilm control around the different dental cavities using bioactive restorative materials able to release F^−^ and Ca^2+^ is an open discussion [26]. Most of the time, the mechanical aspects of these materials are essential to consider them as definitive restorations. However, the results of the study showed that two biomaterials considered bioactive and suitable for long-term restorations can present a different behavior. So, the null hypothesis first formulated has been rejected.

Regarding the acid neutralization property, no significant differences were observed, indicating that both materials were comparable in relation to this property; and, specifically, they exhibited greater action after 7 days of immersion in the buffer solutions. Regarding the effect of pH on ions release, the results showed an increase in the release of F^−^ for both materials in an acid or basic environment, where these ions are most needed. Therefore, on the contrary, a different performance was found with respect to the release of F^−^ and Ca^2+^. In all of the simulated conditions, the ion-releasing percentages were superior for EF than for CN. The explanation can be found in the different classes of dental materials they are part of. CN is a resin-modified composite from the alkasite class that can be used as a self-cured or light-cured material (Table 1). EF is essentially a glass ionomer cement based on an acid-base reaction from a salt compound instead of a polymeric-based composite. On the basis of that, the different chemical and physical characteristics support the visible dissimilar behavior. In fact, EF (high-viscous glass ionomer cement) exhibited a higher ion leaching than CN (alkasite), in all different laboratory conditions (pH and temperature). In a previous study [27], similar to the present investigation, the authors investigated the release of F^−^ from EF and other commercial GICs (glass ionomer cements). They found that the pH was of greater relevance for EF, mainly when no coating varnish was placed on the investigated materials. In the present study, no coat was applied to the samples in order to unaffectedly evaluate the material itself. Previous authors [28] investigated the release of F^−^ and Ca^2+^ and associated this property with the microhardness of 11 ion-leaching restorative materials. They partially confirmed our findings in terms of a higher release of F^−^ and Ca^2+^ for EF than other materials and with respect to CN.

Observing Figures S2 and S3, it is noticeable that the release of F^−^ and Ca^2+^ by the CN varied significantly, increasing after 1 day (1–3 mg/L) to 7 days (1–6 mg/L) and consecutively to 28 days (2–8 mg/L). The T parameter (44, 37, 18, and 0 °C) also significantly influenced the release rates of F^−^ and Ca^2+^, reaching the top at the values of 44°, first, and 37 °C later. This temperature can be assumed to be closer to that of dental tissues, mainly to dentin. Regarding the behavior of EF, it was clear that the release rates of F^−^ and Ca^2+^ were higher (4–30 mg/L) compared to the respective values of CN, reiterating the concept that only after 1 day do the values begin to grow with a higher percentage than the other restoration material.

A restorative material nevertheless needs sufficient strength to be clinically available. A previous study evaluated the clinical performance of both materials evaluated in this investigation. The authors investigated one year of clinical performance between alkasite (Cention N) and high-viscosity glass ionomer (Equia Forte Fil) as restorative materials for Class I cavities. According to their findings, there was no significant difference in clinical performance between the materials tested or between the groups from baseline to 12 months. They concluded that both tested materials showed acceptable clinical performance in the restoration of Class I cavities [29]. Therefore, the present study could be considered complementary to their findings, suggesting that the bioactive aspects support their clinical use under the appropriate conditions. Another clinical study in pediatric dentistry compared the biological, functional, and aesthetic properties of Cention N to glass ionomer cement (GIC) for direct restorations of primary dentition. According to data from their study, both CN materials and GIC showed the same performance after one year [30]. Therefore, the present study corroborates their findings, suggesting the importance of proper curing and handling, as well as the ions’ activity, in controlling biochemical phenomena.

In addition, an in vitro study [31] assessed the evolution of mechanical properties of different ion-releasing restorative dental materials over three months and after accelerated aging in ethanol as well as the water sorption and solubility over one year. It was found that the flexural properties of CN were higher than those of a GIC and lower than those of a conventional resin composite. When left to self-cure, this material exhibited a slow increase in flexural strength and elastic modulus as well as an increase in solubility. On the contrary, when light-cured, CN showed slightly lower values than other light-cured materials in terms of mechanical properties and water sorption. In conclusion, the authors indicated that the alkasite-based restorative material’s mechanical properties are satisfactory and better than light-cured glass ionomer mechanical properties. These phenomena can be related to the existence of a polymeric matrix base in light-curing materials. In the present study, it was confirmed that CN always released a lower percentage of ions (F^−^ and Ca^2+^). The explanation can easily be found in Table 1, where the matrix compositions of the investigated material are introduced. This is in line with the higher base of the UDMA polymeric resin matrix of CN on one side and the missing polymeric structure of the second restorative dental material.

Using artificial caries lesions, it was demonstrated the remineralizing effect of CN restorative material compared with GIC and resin composite in proximal contact and in vitro pH cycling. According to the reported study, CN significantly increased the surface hardness recovery and fluoride content of adjacent enamel caries compared to the composite. In addition, the authors found that the amount of F^−^ in the CN specimens was significantly higher than in the composite specimens but was similar to the GIC specimens [25]. These findings were also confirmed under different conditions investigated [32]. In the present in vitro study, EF efficiently showed the highest ion release values in the medium under different temperatures and pH parameters. EF, a glass ionomer cement, has been more prone to ion release than CN. It remains to understand if this large difference in bioactivity can be really needed in vivo when used as definitive dental restorative materials to replace enamel and dentin lost tissues.

A numerical study evaluated the effect of the combination of different dental filling materials in Class I cavities under occlusal. Two adhesively bonded restorative materials [bulk-fill resin composite (BF) and alkasite (CN)] were evaluated with or without the presence of a base material. According to previous findings, the use of flexible polymeric or ionic base material in combination with bulk-fill resin composite or CN did not reduce the stress magnitude in dentin and enamel. Therefore, adhesively bonded CN restoration showed promising mechanical behavior when used for posterior Class I cavities [21].

It was shown that CN was able to efficiently release F^−^ and Ca^2+^ ions during pH and T variation, considering the usage’s conditions as definitive restorative dental material with adequate strength and rigidity (although this study has not measured any mechanical properties). In this sense, CN could be indicated as an efficient bioactive bulk material of a new class of polymeric filling dental materials [33,34,35] as an alternative to traditional dental resin composite restorative ones [4] in posterior teeth, when high-viscous glass ionomer cements are not available or indicated [36].

## 5. Conclusions

Within the limits of this in vitro investigation, it is possible to conclude:

1. Equia Forte HT filling and Cention N Forte efficiently behave as F^−^ and Ca^2+^ ion releasing dental filling materials.
2. They showed different but stable bioactivity; in this sense, they can contribute to the dental remineralization process and secondary caries prevention.

## Figures and Tables

**Table 1:** Type and composition of the materials investigated in this study.

Commercial Names	Groups	Type	Composition
Cention Forte (Lot n. Z037KP)	CN	Modified composite resin	Calcium fluorosilicate glass, Ba-Al silicate glass, Ca-Ba-Al fluorosilicate glass, ytterbium trifluoride, isofiller UDMA, DCP, aromatic aliphatic-UDMA
Equia Forte HT Fil (Lot n. 211122A)	EF	High-Viscous glass ionomer Cement	Fluoroaluminosilicate glass, polyacrylic acid, iron oxide polybasic carboxylic acid, water

UDMA: urethane dimethacrylate; DCP: tricyclodecan-dimethanol dimethacrylate; Aromatic aliphatic-UDMA: tetramethyl-xylylendiurethane dimethacrylate.

**Table 2:** Mean pH values of soaking water after immersion of the materials in different solutions (acid, neutral, and basic) for three observation periods.

Buffer Solution pH	Material	T (°C)	pH		
			24 h	7 Days	28 Days
4.8	CN	44	5.82 ± 0.05	5.85 ± 0.03	5.63 ± 0.04
		37	5.07 ± 0.03	5.69 ± 0.04	5.46 ± 0.04
		18	4.69 ± 0.06	5.41 ± 0.03	5.23 ± 0.05
		0	4.61 ± 0.03	4.50 ± 0.05	4.36 ± 0.06
	EF	44	5.21 ± 0.07	5.78 ± 0.05	5.32 ± 0.05
		37	4.95 ± 0.05	5.52 ± 0.06	5.27 ± 0.04
		18	4.78 ± 0.04	5.23 ± 0.06	5.11 ± 0.04
		0	4.58 ± 0.05	4.37 ± 0.07	4.78 ± 0.07
6.8	CN	44	5.58 ± 0.07	6.37 ± 0.05	6.20 ± 0.04
		37	5.75 ± 0.03	6.88 ± 0.03	6.49 ± 0.05
		18	5.69 ± 0.05	6.88 ± 0.06	6.61 ± 0.04
		0	5.65 ± 0.05	6.87 ± 0.07	6.69 ± 0.08
	EF	44	5.79 ± 0.06	6.22 ± 0.04	6.31 ± 0.06
		37	5.85 ± 0.04	6.59 ± 0.04	6.43 ± 0.07
		18	5.78 ± 0.05	6.48 ± 0.06	6.77 ± 0.07
		0	5.66 ± 0.04	6.53 ± 0.05	6.82 ± 0.09
8.8	CN	44	6.64 ± 0.07	8.09 ± 0.07	7.85 ± 0.05
		37	7.24 ± 0.08	7.79 ± 0.03	7.53 ± 0.06
		18	7.45 ± 0.05	7.77 ± 0.05	7.54 ± 0.05
		0	7.44 ± 0.06	7.78 ± 0.04	7.60 ± 0.07
	EF	44	6.84 ± 0.04	7.96 ± 0.05	7.94 ± 0.06
		37	7.33 ± 0.03	7.65 ± 0.06	7.65 ± 0.05
		18	7.52 ± 0.05	7.59 ± 0.06	7.62 ± 0.08
		0	7.61 ± 0.03	7.63 ± 0.06	7.58 ± 0.07

**Table 3:** Average fluoride ion concentration released according to the material, pH conditions (acid, neutral, and basic), observation time (24 h, 7 days, and 28 days), and temperature (0, 18, 37, and 44 °C).

Buffer Solution pH	Time	T (°C)	Fluoride Ion Release Mean ± SD (mg L^−1^)	
			CF	EF
4.8	24 h	44	1.49 ± 0.17	1.46 ± 0.23
		37	0.69 ± 0.15	0.99 ± 0.19
		18	0.19 ± 0.01	0.38 ± 0.02
		0	0.16 ± 0.03	0.11 ± 0.02
	7 days	44	2.76 ± 0.36	14.62 ± 0.42
		37	4.30 ± 0.33	28.71 ± 1.20
		18	0.27 ± 0.03	9.99 ± 0.99
		0	0.25 ± 0.04	10.68 ± 0.53
	28 days	44	5.27 ± 0.36	23.81 ± 1.11
		37	3.95 ± 0.23	29.62 ± 0.66
		18	4.39 ± 0.44	12.47 ± 0.76
		0	0.38 ± 0.03	11.16 ± 0.56
6.8	24 h	44	1.27 ± 0.19	1.48 ± 0.26
		37	0.44 ± 0.08	1.46 ± 0.26
		18	0.22 ± 0.05	1.55 ± 0.27
		0	0.16 ± 0.04	0.37 ± 0.07
	7 days	44	1.33 ± 0.18	12.88 ± 0.66
		37	2.54 ± 0.34	13.95 ± 0.36
		18	2.45 ± 0.34	13.11 ± 0.38
		0	1.05 ± 0.12	5.43 ± 0.39
	28 days	44	7.95 ± 0.69	15.94 ± 0.91
		37	4.13 ± 0.37	16.16 ± 0.63
		18	7.15 ± 0.60	13.09 ± 0.68
		0	1.14 ± 0.21	6.06 ± 0.57
8.8	24 h	44	1.06 ± 0.14	1.70 ± 0.25
		37	0.65 ± 0.14	3.63 ± 0.25
		18	0.30 ± 0.06	1.39 ± 0.27
		0	0.16 ± 0.03	1.10 ± 0.18
	7 days	44	1.64 ± 0.25	14.86 ± 0.26
		37	5.44 ± 0.57	29.34 ± 0.84
		18	3.86 ± 0.23	14.61 ± 0.58
		0	1.38 ± 0.26	11.03 ± 0.60
	28 days	44	10.08 ± 0.62	28.62 ± 1.19
		37	7.85 ± 0.51	32.56 ± 1.11
		18	6.07 ± 0.24	14.32 ± 0.48
		0	1.48 ± 0.16	12.11 ± 0.54

SD: Standard deviation (n = 5).

**Table 4:** Average concentration of calcium ions released from both materials for pH = 4.8, 6.8, and 8.8, at three observation times (24 h, 7 days, and 28 days) and four temperatures (0, 18, 37, and 44 °C).

Buffer Solution pH	Time	T (°C)	Calcium Ion Release Mean ± SD (mg L^−1^)	
			CN	EF
4.8	24 h	44	2.09 ± 0.28	4.34 ± 0.40
		37	1.30 ± 0.24	2.75 ± 0.27
		18	0.42 ± 0.05	0.99 ± 0.15
		0	0.20 ± 0.03	0.56 ± 0.08
	7 days	44	8.27 ± 0.46	21.64 ± 0.67
		37	12.58 ± 0.43	18.17 ± 0.71
		18	2.56 ± 0.13	2.98 ± 0.13
		0	0.73 ± 0.11	4.69 ± 0.51
	28 days	44	10.47 ± 0.70	22.99 ± 0.66
		37	14.31 ± 0.74	18.99 ± 1.27
		18	5.02 ± 0.31	3.06 ± 0.26
		0	1.84 ± 0.13	4.91 ± 0.23
6.8	24 h	44	2.66 ± 0.18	1.63 ± 0.31
		37	0.74 ± 0.09	5.48 ± 0.54
		18	0.18 ± 0.02	0.50 ± 0.05
		0	0.16 ± 0.04	0.27 ± 0.03
	7 days	44	6.22 ± 0.17	8.11 ± 0.83
		37	5.97 ± 0.62	25.94 ± 0.84
		18	2.17 ± 0.28	5.00 ± 0.21
		0	0.21 ± 0.02	1.72 ± 0.27
	28 days	44	3.73 ± 0.49	10.77 ± 0.91
		37	6.30 ± 0.47	29.57 ± 0.60
		18	2.12 ± 0.21	5.19 ± 0.40
		0	0.30 ± 0.04	2.55 ± 0.13
8.8	24 h	44	1.83 ± 0.27	3.81 ± 0.44
		37	0.93 ± 0.07	2.33 ± 0.26
		18	0.17 ± 0.03	1.49 ± 0.32
		0	0.15 ± 0.04	0.46 ± 0.03
	7 days	44	12.94 ± 0.59	10.40 ± 0.40
		37	9.08 ± 0.42	11.37 ± 0.81
		18	2.98 ± 0.47	7.11 ± 0.71
		0	0.13 ± 0.03	3.90 ± 0.33
	28 days	44	13.35 ± 0.76	11.23 ± 1.35
		37	11.75 ± 1.00	11.88 ± 0.91
		18	2.36 ± 0.27	7.61 ± 0.68
		0	0.18 ± 0.01	3.92 ± 0.74

SD: Standard deviation (n = 5).

## Data Availability

Not applicable.

## Supplementary Materials

The following supporting information can be downloaded at: https://www.mdpi.com/article/10.3390/jfb14020109/s1, Figure S1: pH changes after three observation times (24 h, 7 and 28 days) and four temperatures (0, 18, 37 and 44 °C) in 3 different buffered solution: (a) pH = 4.8; (b) pH = 6.8 and (c) pH = 8.8; Figure S2. Average concentration of fluoride ion released from Materials 1 and 2 for three observation times (24 h, 7 and 28 days) and four temperatures (0, 18, 37 and 44 °C) in (a) acid medium (pH = 4.8); (b) neutral environment (pH = 6.8); (c) basic environment (pH = 8.8); Figure S3. Average concentration of calcium ion released from the Material 1 and 2 for three observation times (24 h, 7 and 28 days) and four temperatures (0, 18, 37 and 44 °C) in (a) acid medium (pH = 4.8); (b) neutral environment (pH = 6.8); (c) basic environment (pH = 8.8).
