# Supplementary material for: Fluoride and Calcium Release from Alkasite and Glass Ionomer Restorative Dental Materials: In Vitro Study
Source: J Funct Biomater. 2023 Feb 15;14(2):109. doi: 10.3390/jfb14020109 (PMC9967494; doi:10.3390/jfb14020109)
Supplement: Supplementary file 1 [file jfb-14-00109-s001.zip › jfb-2183114-supplementary.pdf]

Journal of *Functional Biomaterials*

MDPI

SUPPLEMENTARY MATERIALS

Fluoride and Calcium Release from Alkasite and Glass Ionomer Restorative Dental Materials: In-Vitro Study

Alessandro di Lauro [1], Fabiana Di Duca [2,*], Paolo Montuori [2], João Paulo Mendes Tribst [3], Amanda Maria de Oliveira Dal Piva [4], Alexandre Luiz Souto Borges [5] and Pietro Ausiello [1]

Figure 1S. pH changes after three observation times (24 h, 7 and 28 days) and four temperatures (0, 18, 37 and 44 °C) in 3 different buffered solution: **a**) pH = 4.8; **b**) pH = 6.8 and **c**) pH = 8.8.

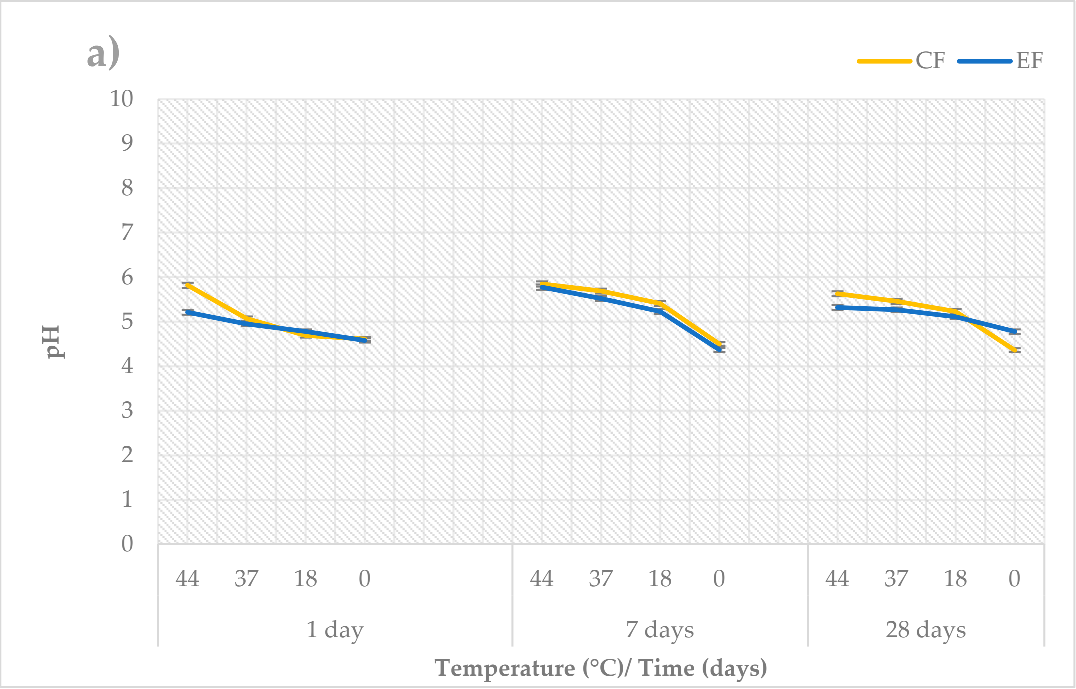

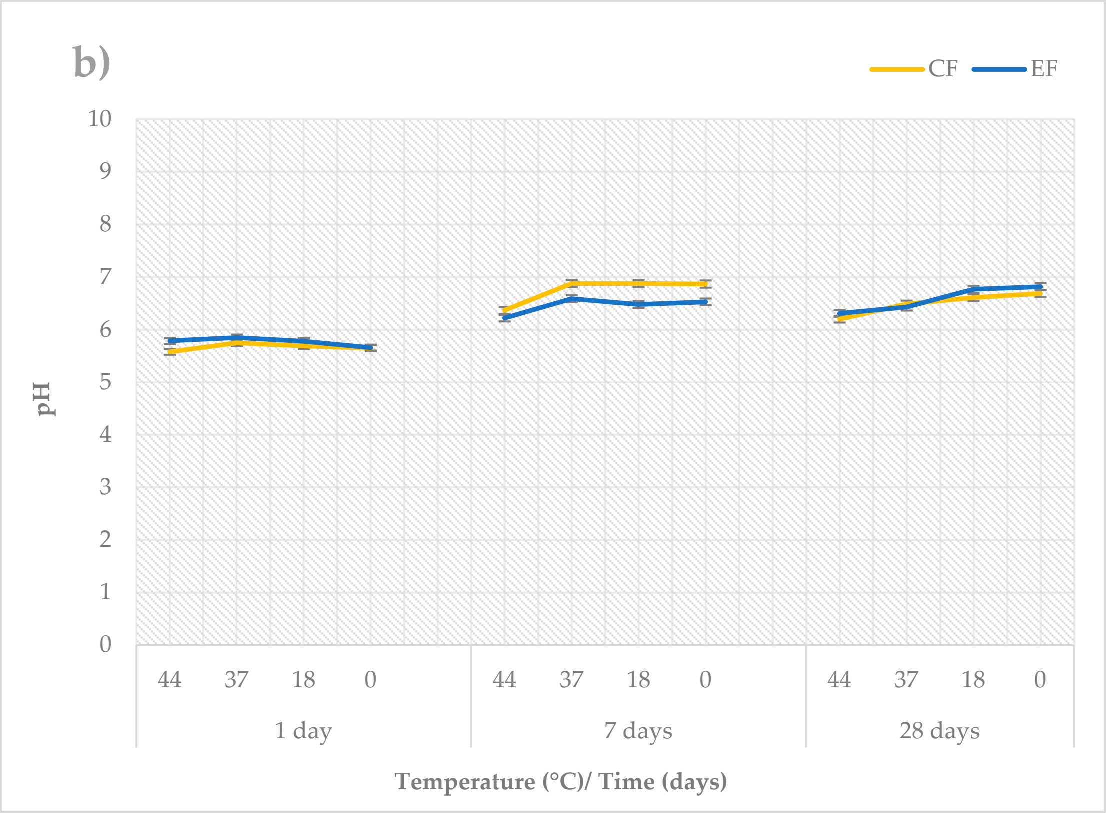
b)
CF
EF
pH
10
9
8
7
6
5
4
3
2
1
0
44
37
18
0
1 day
7 days
28 days
Temperature (°C)/ Time (days)

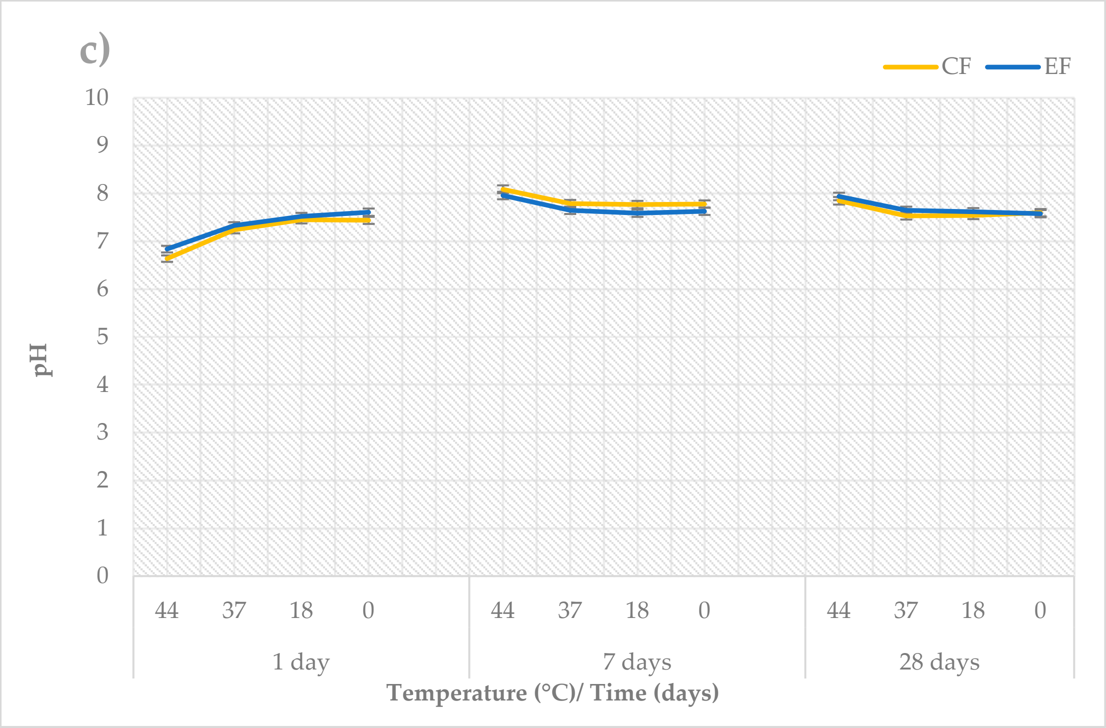
c)
CF
EF
pH
10
9
8
7
6
5
4
3
2
1
0
44
37
18
0
1 day
7 days
28 days
Temperature (°C)/ Time (days)

Figure 2S. Average concentration of fluoride ion released from Materials 1 and 2 for three observation times (24 h, 7 and 28 days) and four temperatures (0, 18, 37 and 44 °C) in **a**) acid medium (pH = 4.8); **b**) neutral environment (pH = 6.8); **c**) basic environment (pH = 8.8).

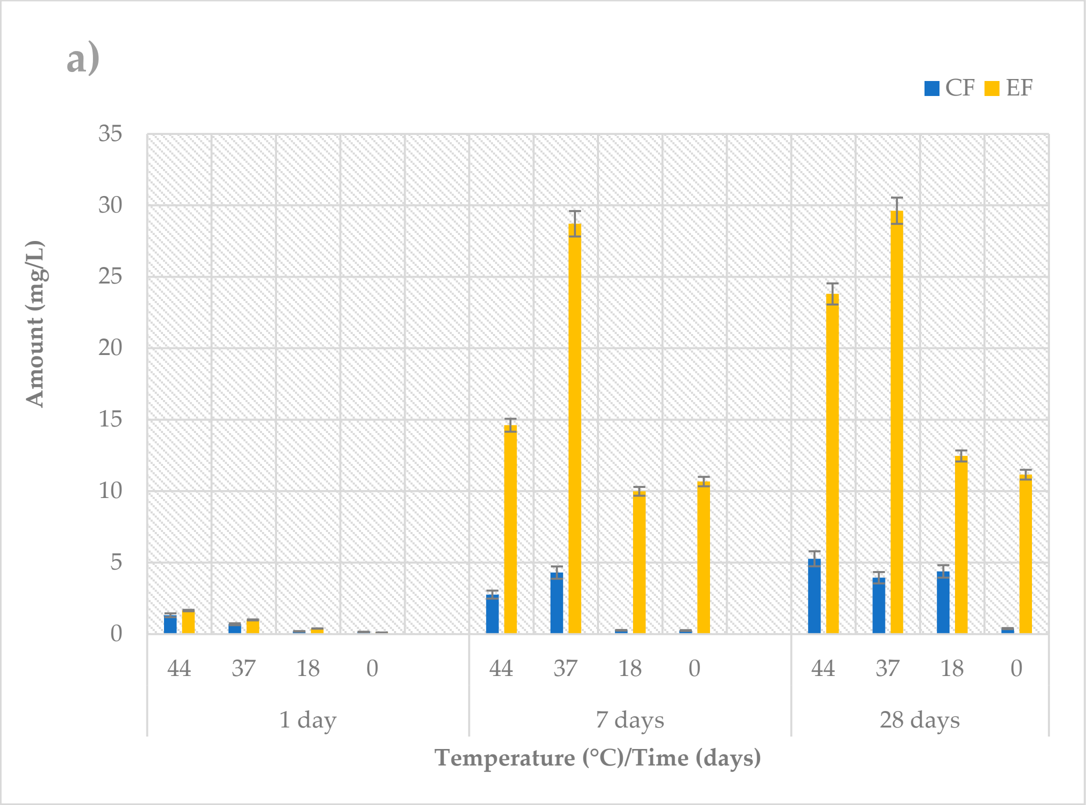

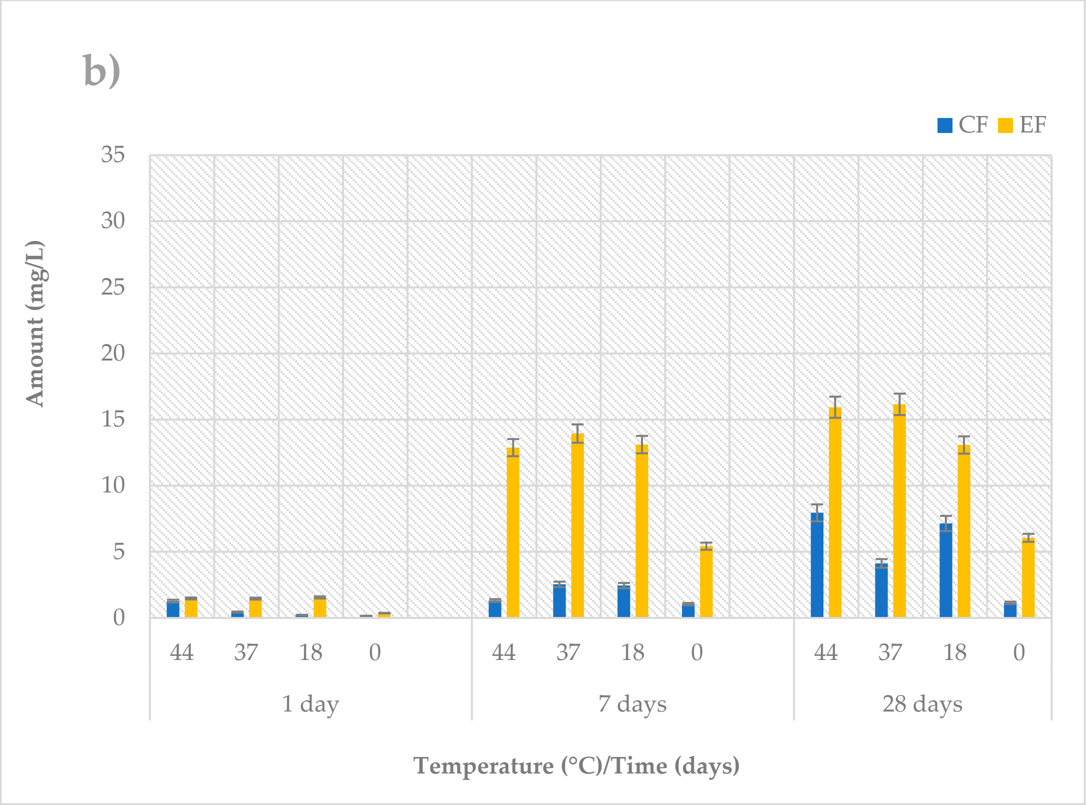

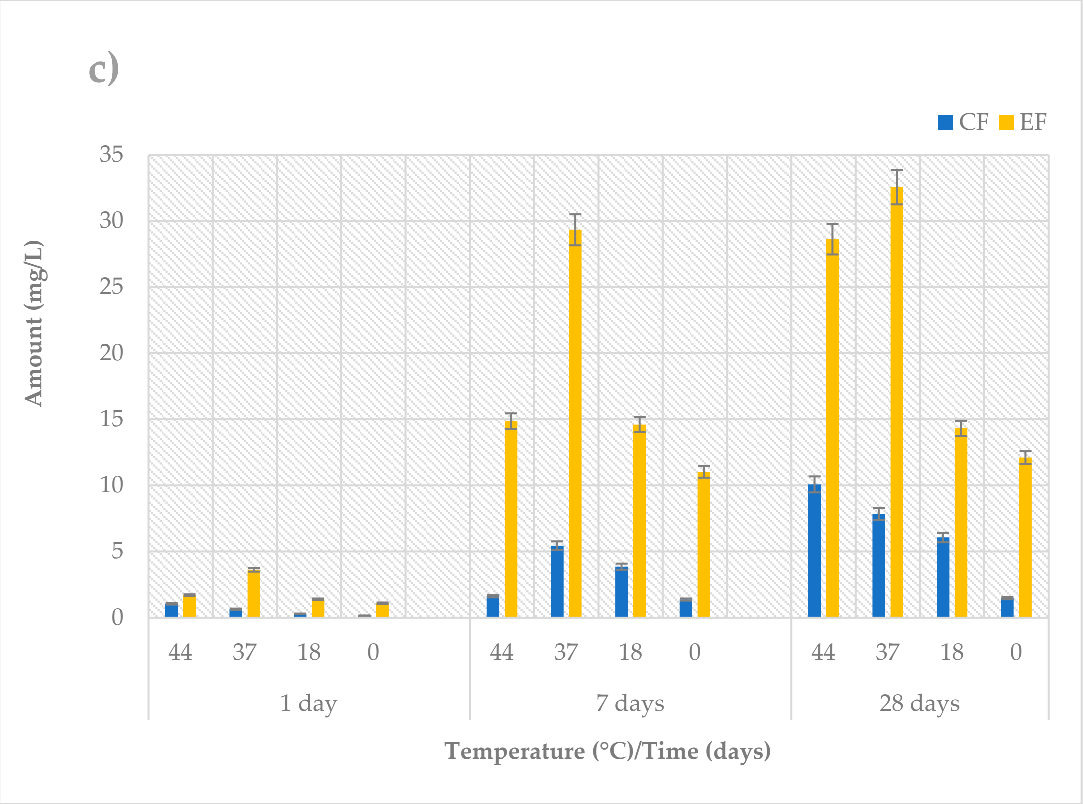
c)
CF EF
35
30
25
20
15
10
5
0
Amount (mg/L)
44 37 18 0
1 day
44 37 18 0
7 days
44 37 18 0
28 days
Temperature (°C)/Time (days)

Figure 3S. Average concentration of calcium ion released from the Material 1 and 2 for three observation times (24 h, 7 and 28 days) and four temperatures (0, 18, 37 and 44 °C) in **a**) acid medium (pH = 4.8); **b**) neutral environment (pH = 6.8); **c**) basic environment (pH = 8.8).

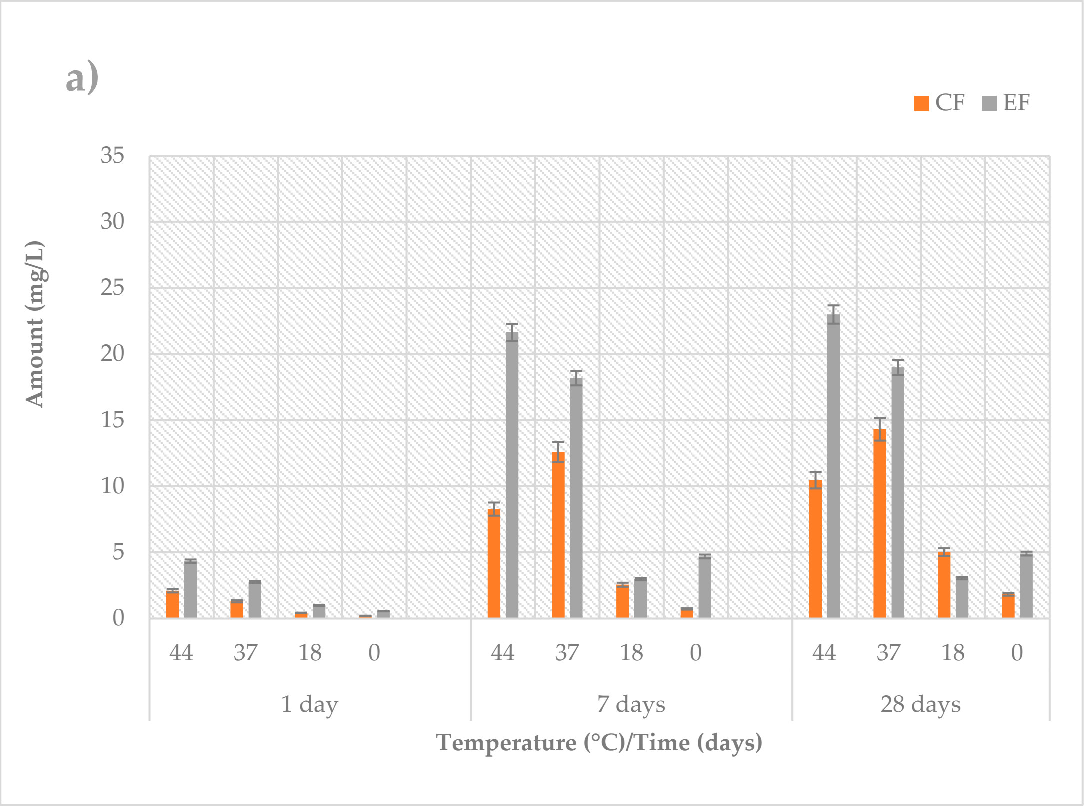

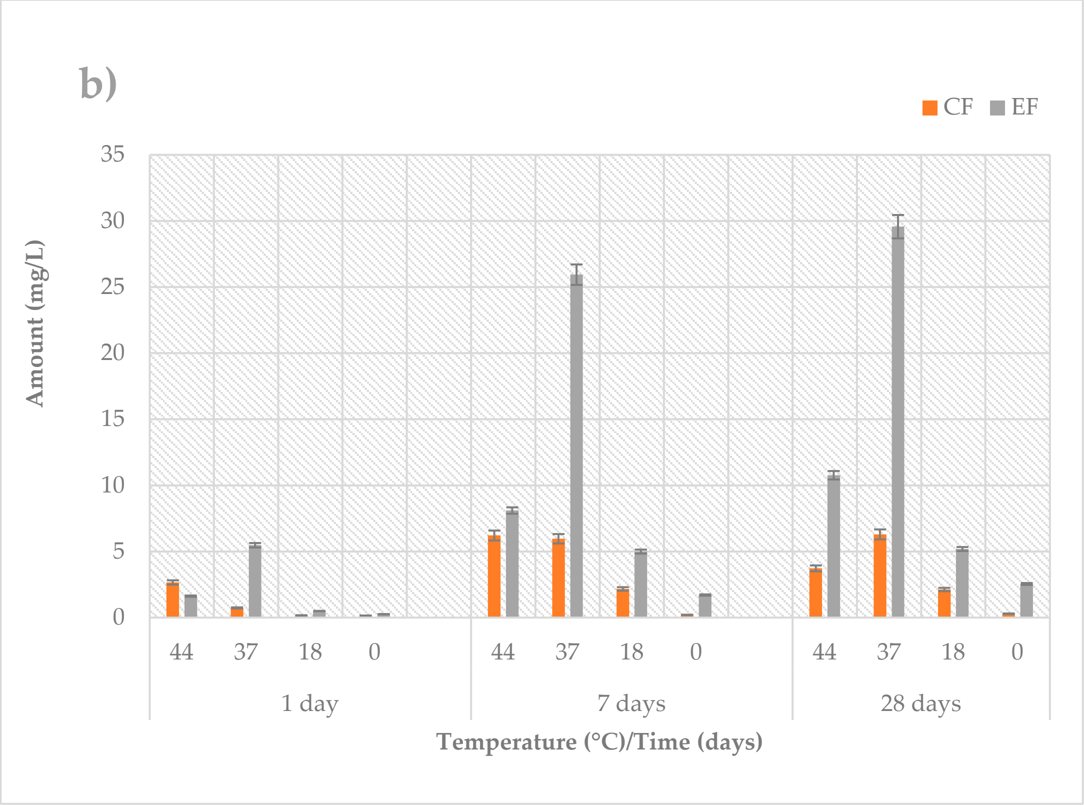

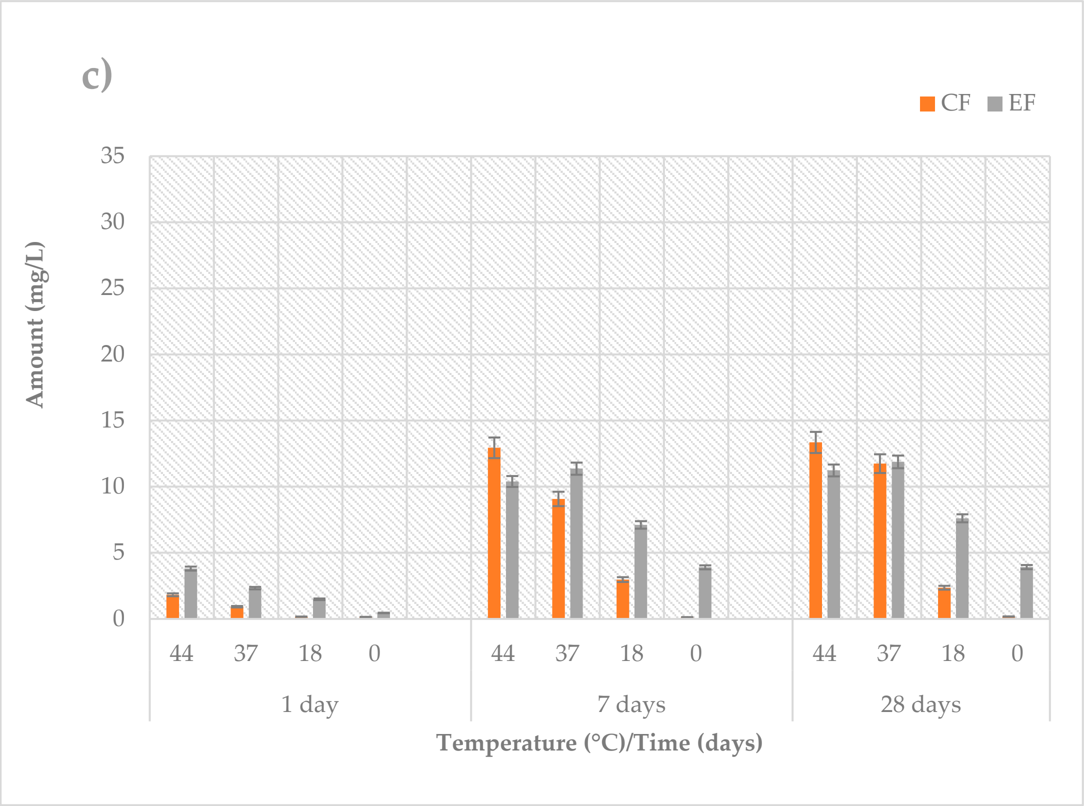
c)
CF
EF
35
30
25
20
15
10
5
0
Amount (mg/L)
44
37
18
0
44
37
18
0
44
37
18
0
1 day
7 days
28 days
Temperature (°C)/Time (days)
